# Polygenic anti-cancer activity of *Indigofera macrophylla* in prostate cancer induced animal model

**DOI:** 10.1016/j.toxrep.2024.101774

**Published:** 2024-10-22

**Authors:** Gbenga Oluwaseyi Alabi, Olusola Olalekan Elekofehinti, David Morakinyo Sanni, Joseph Opeolu Ashaolu, Adedotun Olayemi Oluwatuyi

**Affiliations:** a Bioinformatics and Molecular Biology Unit, Department of Biochemistry, Federal University of Technology Akure, Ondo State, Nigeria; b Enzymology and Molecular Biology Unit, Department of Biochemistry, Federal University of Technology Akure, Ondo State, Nigeria; c Department of Biochemistry, Faculty of Basic Medical Sciences, Redeemers University, PMB 230, Osun State, Nigeria; d Teady Bioscience Research Laboratory, 42, Adinlewa Street, Akure, Ondo State, Nigeria

**Keywords:** Prostate cancer, Inflammatory, Apoptotic, Genes, *Indigofera macrophylla*

## Abstract

**Background:**

Prostate cancer (Pca) is a deadly disease prevalent among men, and it accounts for about 7–8 % of mortality globally. Synthetic drugs have proved effective but have limitations and severe side effects. There is, therefore, a need to discover a less expensive, natural therapeutic agent with no side effects in treating the ailment.

**Aim:**

The study aims to investigate the anti-prostate cancer activity of extracts of *Indigofera macrophylla* (*I. macrophylla*) at the physiological and molecular levels in experimental animals.

**Method:**

Polyphenol-rich extract of *I. macrophylla* was subjected to HPLC analysis to identify the plant's phytochemical constituent. Adult Wistar rats were orally administered 2mls of 50, 100 and 200 PPM of the cacodylic acid solution for 28 days to induce prostate cancer, while treatment was carried out by orally administering extract of *I. macrophylla* at doses of 50, 100 and 200 mg/kg for up to 28 days. The anti-inflammatory and apoptotic properties of the extract in experimental animals were investigated by the expression levels of various genetic biomarkers such as Bax-2, TNF-α, IL-6, COX2, IL-1β, β-Catenin, APC, Bcl2, CEA, Caspase 3 and β-Catenin using reverse transcriptase polymerase chain reaction (RT-PCR).

**Result:**

HPLC analysis shows that *I. macrophylla* has 21 bioactive components which are categorized into seven groups: flavonoid, terpenes, phenols, isoflavonoid, phytosterols, quinone and glycosides. Administration of the drug shows inconsistencies in the mean body weights of the experimental animals. Further investigation revealed that *I. macrophylla* increased TNF-α upregulation and expression, significantly downregulated IL-1β, significantly decreased IL-6 expression, ameliorated COX2 expression, downregulated β-catenin expression and significantly reduced the expression of the APC gene. These results show that the drug activity modulates the investigated inflammatory and apoptotic genes in the prostate gland of PCa-induced rats, thus demonstrating its anti-PCa potential.

**Conclusion:**

The results of this study suggest the potential of a novel treatment protocol of *I. macrophylla* plant extract to improve therapeutic outcomes for patients with aggressive PCa, which reportedly claims hundreds of thousands of lives yearly.

## Highlights

• Prostate cancer (Pca) a deadly disease prevalent among men.
• Synthetic drugs have proved effective but have limitations and severe side effects.
• *Indigofera macrophylla* is a Polyphenol-rich extract with high anticancer properties.
• Gene expression level investigation for anti-inflammatory and apoptotic properties.

## Graphical Abstract

**Figure:**
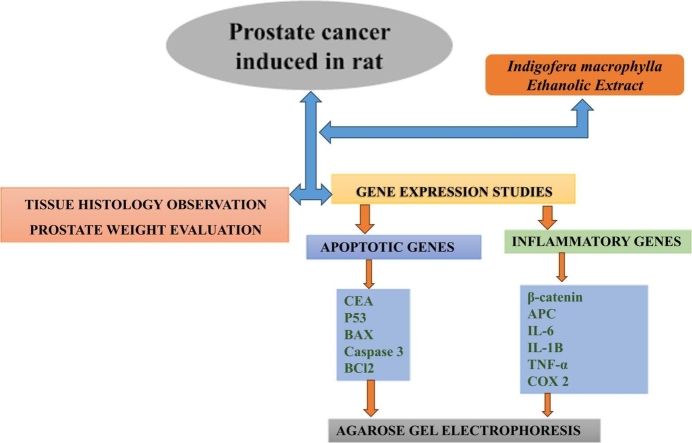

## 1 Introduction

The development of malignant tumour in males’ prostate gland of the reproductive system is referred to as prostate cancer (PCa) [1]. Various data suggest that PCa remains one of the leading causes of death globally as it is reportedly the most frequent cancer among men in Western and developing nations, especially in persons of African heritage [2]. About 899,000 cases and 258,000 deaths worldwide are attributed to PCa each year. The prognosis for PCa, a complex and heterogeneous disease, varies. PCa can progress more aggressively and become a castration-resistant illness if left untreated [3], [4]. According to a study by Pham-Huy *et al*., the main causes of PCa are free radicals and related reactive species produced by certain lifestyle choices, such as smoking, drinking alcohol, eating much meat, and eating little vegetables. As a result, there is much interest in preventive and therapeutic measures against the development of PCa [5].

Currently, chemotherapy, radiation therapy, and surgery are used for the treatment of PCa. Steroids (such as cyproterone acetate) and nonsteroidal androgen antagonists (such as bicalutamide enzalutamide) are among the therapeutic drugs frequently utilized in the initial line of PCa treatment [6]. Due to their partial agonistic activities and impacts on other hormonal systems, steroids can cause gynecomastia, erectile dysfunction, loss of libido, and major cardiovascular issues, among other concerns [7]. Although non-steroidal anti-androgens have a higher oral bioavailability than steroidal anti-androgens, they nonetheless have several adverse effects [8]. Besides, these PCa treatments are costly and prone to resistance. Therefore, one of the main goals of researchers in the field of current cancer drug discovery is to find an alternative that is accessible, inexpensive, effective, and less hazardous.

Over the past 30 years, there has reportedly been a massive increase in usage of herbal products and supplements. It is believed that 80 % of the world's population depends on them for some form of primary healthcare [9]. Plant parts, including seeds, fruits, leaves, roots, stems, bark, or flowers, can all be used as therapeutic cures when making herbal medications. Numerous medicinal plants have been shown to have antioxidant, anticancer, and antitumor effects in earlier research [10], [11]

One of such plants whose therapeutic properties have been reported *is Indigofera macrophylla*. The genus Indigofera, comprising approximately 750 species, is widely used in traditional medicine for various ailments [12]. Phytochemical studies have identified over 200 compounds in the Indigofera species, primarily flavonoids and terpenoids [12]. These plants exhibit diverse pharmacological activities, including antimicrobial, cytotoxic, and anti-inflammatory effects [12], [13]. Specific Indigofera species, such as *I. colutea, I. tinctoria, I. nigritana, and I. macrocalyx,* have demonstrated significant antioxidant properties, correlating with their high phenolic content [14]. The alkaloid indirubin, isolated from Indigofera, shows promising therapeutic potential and is currently undergoing clinical trials [12]. More so, high antimicrobial activities of *I. daleoides*, *I. dendroides, I. longeraceae, I. and oblingifolia* [15], [16], [17], [18] has been reported. Pharmacological research has also demonstrated the diverse range of qualities exhibited by crude extracts as well as purified fractions of Indigofera species, such as anti-arthritic [19], antibacterial [20], and anticancer [21] capabilities. Despite the genus's wealth of study, there are still unanswered questions about the pharmacology and phytochemistry of some species, such as *I. macrophylla*, particularly the mechanisms underlying the actions of its active chemicals [12]. The widespread use of this plant, coupled with anecdotal evidence and pharmacological properties, led to investigations on its anti-PCa potential. Hence, this study aimed to investigate the ameliorative effectiveness of aqueous leaf extract of *I. macrophylla* on some selected apoptotic and inflammatory genes implicated in PCa development in experimentally PCa-induced rats.

## 2 Material and method

### 2.1 Plant materials and extraction procedure

In July 2021, fresh *I. macrophylla* leaves were gathered from Ile-Ife in the state of Osun. At the Federal University of Technology, Akure, the sample was recognized and verified using identification/herbarium number 0302. *I. macrophylla* leaves were air-dried, rinsed with clean water, and crushed into a powder. 2.5 litres of pure ethanol were used to soak 800 g of powdered *I. macrophylla* leaves. Whatman No. 4 filter paper was used to filter the mixture. Using a rotary evaporator set to 175 mbar of pressure and 40°C temperature, the filtrate produced was concentrated into a slurry. It was then freeze-dried directly into a fine powder and kept in a sealed container until needed. With a little adjustment, phenolics were extracted using the method outlined by Ejelonu *et al*. [22]. The source of cacodylic acid was Nanjing Xingang, China. Additional chemicals and reagents were bought from a few regular Nigerian suppliers, such as Santa Cruz Biotechnology, Sigmal-Adrich, and Inqaba Biotech.

### 2.2 HPLC determination ethanolic extract of *I. macrophylla.*

Different extract batches were tracked using chromatographic fingerprinting. The samples were analyzed using a Shimadzu HPLC system (Kyoto, Japan). The UltimateXB-C18 column's absorbance (150_4.6 mm2, 5 μm) was determined at 28 nm. In the mobile phase, acetonitrile (ACN) was used as solvent B and water as solvent A. 10 µl was the injection volume, while 0.5 ml/min was the flow rate. After a gradient process, the acetone concentrations were 10–10 % at 0–5 min, 10–85 % at 5–60 min, and 85–85 % at 60–65 min. The most reliable technique for producing this extract was identified by analyzing the best fingerprint.

### 2.3 Animals

For this investigation, 42 Wistar male rats weighing between 150 and 240 g were used. The Animal Ethics Committee of the FUT, Akure School of Science, authorized all procedures about animal studies. For 14 days, the animals were kept in room-temperature housing. They were given constant access to commercial pellets, rat feed (Standard Growers, Grand Cereals LTD, Ibadan, Nigeria) and water. The ingredients of the rat diet include methionine, bone meal, lysine, limestone, salt, vitamins/minerals premix, antioxidants, groundnut cake, soya cake, full-fat soya, brewer dried grains, palm kernel meal, rice bran, and soya [23].

### 2.4 Prostate cancer induction

Forty-two (42) rats were divided into seven (7) groups of six (6) rats in each group. Oral administration of cacodylic acid was used for the induction of prostate cancer. Group I was the control; groups II and III were induced with 50PPM; groups IV and V were induced with 100PPM, and groups VI and VII were given 200PPM of cacodylic acid solution through oral gavage. The animals were orally induced with 2mls of the concentrations mentioned above of cacodylic acid for 28 days. PSA levels of the rats were determined to confirm the successful induction of the disease. The care and use of animals in this study were conducted strictly with institutional and national committee requirements [23].

### 2.5 Treatment of prostate cancer

The rats (n = 42) were divided into seven groups for treatment. Group I served as control and were given 2 ml distilled water by gavage; group II served as prostate cancer control (cancer induced but not treated); group III was prostate cancer-induced and treated with standard drugs (finasteride); groups IV-VI were prostate cancer-induced rats treated with IM plant extracts at doses of 50, 100 and 200 mg/kg body weight respectively while group VII was control rat given 200 mg/kg plant extract only. Oral delivery (treatment) was carried out once a day using a cannula for 28 days. The leaf extract proved safe even at 5000 mg/kg (from a preliminary LD50 study conducted in our laboratory, study not published), which informs our choice of concentration for the experiment. The weights of experimental animals were measured with an electronic weighing balance at the start of the experiment and subsequently once a week for four weeks. Additional weight gains or loss in animals were calculated by deducting the starting weight from the final weight. After treatment, animals were deprived of food and water overnight and euthanized under anaesthesia using halothane before they were sacrificed. Blood samples and tissues of prostate glands were collected for analysis.

### 2.6 Tissue histology

Drury *et al*. [24] method was used to conduct the histopathological analysis of the prostate tissues from the experimental animals. For about 48 hours, the tissues were preserved in 10 % phosphate-buffered formalin. Using conventional methods, they were made ready for histopathological analysis. A light microscope (Motic®) was used to examine the slides, and a Motic® microscope camera was used to take the photos.

### 2.7 Study of gene expression

Elekofehinti *et al*. [25] approach was followed for conducting the gene expression investigation. Prostate tissues that had been trizol-preserved were mechanically homogenized to allow for complete exposure of the cell nucleus. Zymo Quick-RNA™ MiniPrep Kit (Zymo Research) was used for total RNA extraction following the manufacturer’s protocol. UV–VIS spectrophotometer was used to assess the quality and quantity of RNA extracted at 260 and 280 nm absorbance, respectively.

### 2.8 cDNA synthesis

The reverse transcriptase polymerase chain reaction (RT-PCR) was used to convert the obtained total RNA into complementary DNA (cDNA). The conversion of RNA to cDNA was started by the enzyme reverse transcriptase [26]. Following cDNA synthesis, the target genes were amplified using a set of forward and reverse primers carefully designed and optimized (see Table 1). For thirty cycles, the amplification is catalyzed by the PCR Master Mix using a thermocycler (Eppendorf Mastercycler AG 22,331) Hamburg.

**Table 1:** A list of primers specifically created, optimized, and manufactured for each gene.

Gene	Sequence
GADPH	Forward: AGACAGCCGCATCTTCTTGT Reverse: CTTGCCGTGGGTAGAGTCAT
β Catenin	Forward: CCTCTATGCCAACACAGTGC Reverse: CATCGTACTCCTGCTTGCTG
Bcl−2	Forward: GTATGATAACCGGGAGATCG Reverse: AGCCAGGAGAAATCAAACAG
Bax	Forward: AAGAAGCTGAGCGAGTGTCT Reverse: CAAAGATGGTCACTGTCTGC
P53	Forward: ACATGACTGAGGTCGTGAGA Reverse: GATTTCCTTCCACCCGGATAAG
Caspase	Forward: TGTATGCTTACTCTACCGCACCCG69 Reverse: TGTATGCTTACTCTACCGCACCCG
COX−2	Forward: GATTGACAGCCCACCAACTT Reverse: CGGGATGAACTCTCTCCTCA
Interleukin 6 (IL−6)	Forward: CCGGATGGGTAGGATAAAGTT Reverse: ACCCACTGAGGTAGGAAAGA
Interleukin 1β (IL−1β)	Forward: TCTCTCCGCAAGAGACTTCCA Reverse: ATACTGGTCTGTTGTGGGTGG
Tumour necrosis factor alpha (TNF-α)	Forward: ACCACGCTCTTCTGTCTACTG Reverse: CTTGGTGGTTTGCTACGAC

### 2.9 Agarose gel electrophoresis

To allow the amplicons to migrate from the anode to the cathode at a constant voltage, the RT-PCR product amplicons were electrophoresed on 1 % agarose gel. Using Image J software, the relative density and intensity of the gene bands were measured.

### 2.10 Analytical statistics

The results were expressed using the means for each group. GraphPad Prism version 8.0 (GraphPad Software Inc., CA, and USA) was used to analyze the data. One-way analysis of variance (ANOVA) was used to evaluate the data; a post hoc test for multiple comparisons was used to compare group means, which were expressed as mean ± standard error of the mean (SEM), with p values less than 0.05 (p˂0.05) considered statistically significant.

## 3 Results

### 3.1 Phytochemical screening of *I. macrophylla* leaf extract

Based on a phytochemical analysis, *I. macrophylla* extract contained 21 bioactive compounds. These compounds include rutin, myricetin, chrysin, hesperidin, genistein, alpha-caryophyllene, stigmasterol, purpurin, alpha-amylin, methoxy purpurin, indican, ferulic acid, coumestrol, kaempferol, gallic acid, caffeic acid, ellagic acid, chlorogenic acid, quercetin, catechins, and indigoferone. These compounds are categorized into seven major categories of phytochemicals, respectively. Table 2 shows the principal categories: phenols, flavonoids, isoflavonoids, terpenes, phytosterols, quinones, and glycosides.

**Table 2:** Phytochemical constituent of *I. macrophylla* in comparison with other studied species in the genus.

S/n	Main Phytochemical groups	Phytochemical Compounds	Previous screening			
			*Indigofera Linifolia*[27]	*Indigofera tinctoria* [28]	*Indigofera hochstetteri* [29]	*Indigofera longeracemosa* [30]
1.	Phenols	Ferulic acid, Ellagic acid, Gallic acid, Chlorogenic acid, Caffeic acid, Coumestrol	-	+	+	-
2.	Flavonoids	Kaemferol, Quercetin, Catechins, Indigoferone, Rutin, Myricetin, Chrysin, Hesperidin	+	+	+	+
3	Isoflavonoids	Genistein	-	+	+	-
4	Terpenes	Alpha-Caryophyllene	+	+	-	-
5	Phytosterols	Stigmasterol	-	-	-	-
6	Quinones	Purpurin, Methoxypurpurin	-	+	-	-
7	Glycosides	Indican, Rutin	+	+	+	-

### 3.2 Effect of oral administration of cacodylic acid on mean body weight

The effects of oral administration of cacodylic acid are illustrated in Fig. 2a. There is a slight increase, non-significant howbeit, in the body weight of the control (group 1), whereas groups 2–7 exhibit a significant increase in the mean body weight (*p* < 0.05) of experimental animals.

**Fig. 1:**
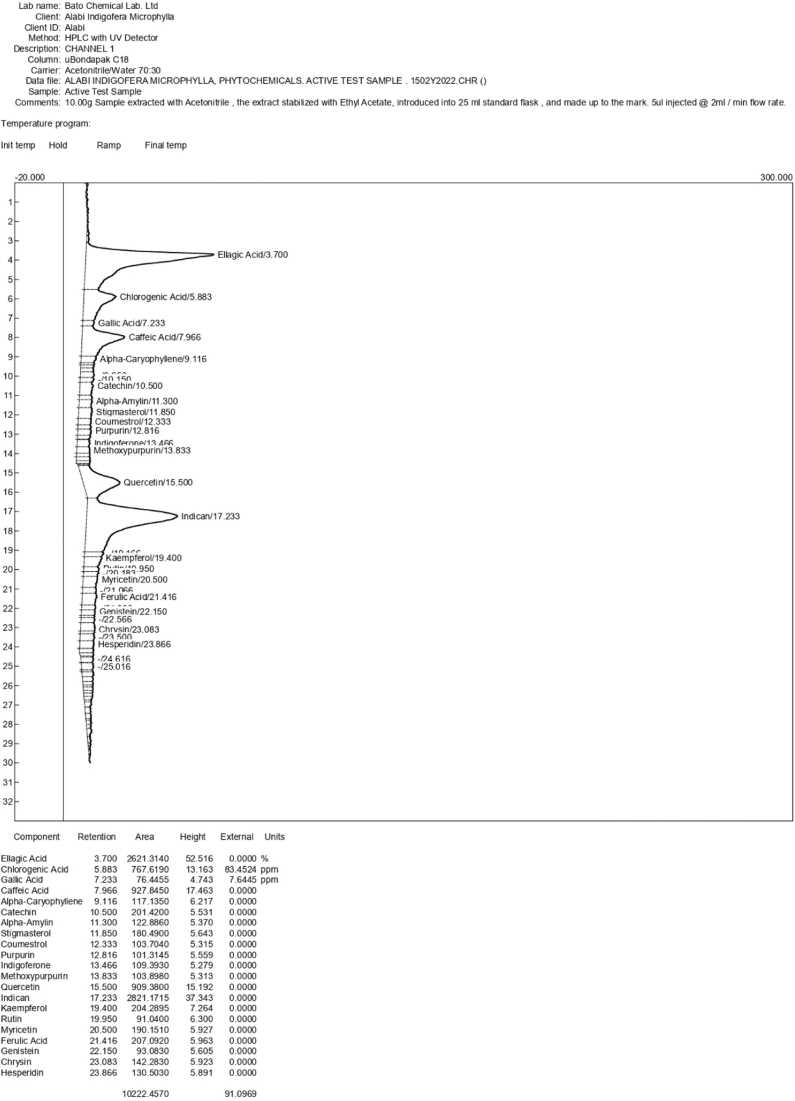
A pictorial diagram showing the HPLC results and the compounds involved.

**Fig. 2:**
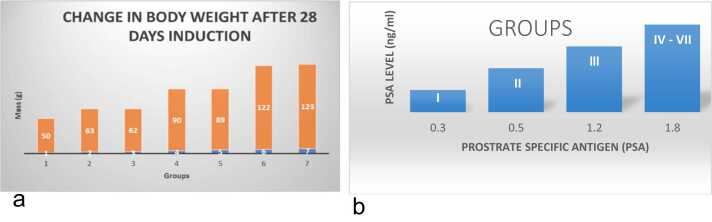

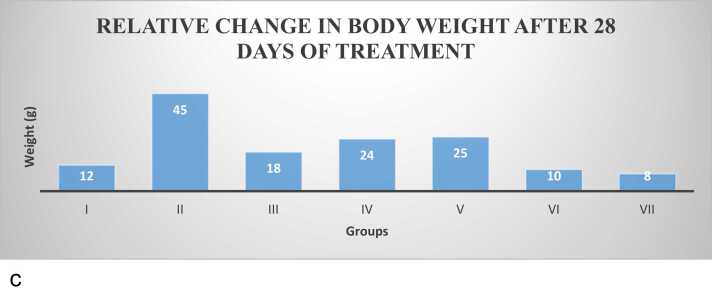
a: Comparative effect of cacodylic acid on the mean body weight of prostate cancer induced rats. Data are expressed as mean ± SEM. Fig. 2b: Comparative effect of cacodylic acid on the PSA level of the induced rats, after 28 days oral Cacodylic acid induction. Fig. 2c: Comparative effect of *I. macrophylla* on relative change in body weight after 28days of treatment of prostate cancer induced rats. Data are expressed as mean ± SEM (*n* = 6); ∗*p <* 0.05; ∗∗*p <* 0.01; ∗∗∗*p <* 0.001; ∗∗∗∗*p <* 0.0001; ∗denotes that data were compared with Induced group.

**Fig. 3:**
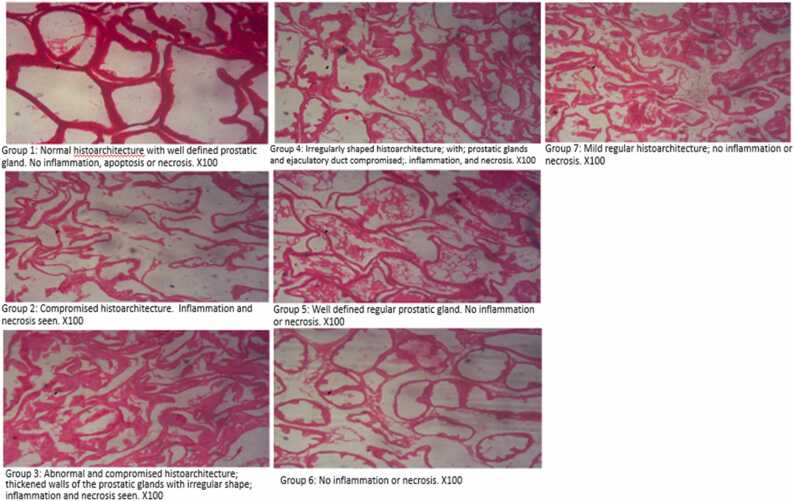
Pictographs of histopathological examinations of the experimental animals by groups.

### 3.3 Effect of oral administration of cacodylic acid on rat PSA level

The effects of oral administration of cacodylic acid on PSA levels are illustrated as a bar chart in Fig. 2b. There is a significant increase in the PSA level of groups 4–7 (p < 0.05), which are induced with 100PPM and 200PPM, respectively.

Effect of oral administration of *I. macrophylla* on mean body weight

### 3.4 Effect of oral administration of *Indigofera macrophylla* on mean body weight

The effects of oral administration of *I. macrophylla* (Fig. 2c) showed a slight increase in the body weight of the control (group I). At the same time, there was a significant increase in the mean body weights of group II (*p* < 0.05) induced with prostate. There were no significant changes in the mean body weight of group III (with standard drug-Finasteride) and groups IV, V, VI and VII.

### 3.5 Effect of oral administration of *I. macrophylla* on inflammatory genes

**Fig. 4:**
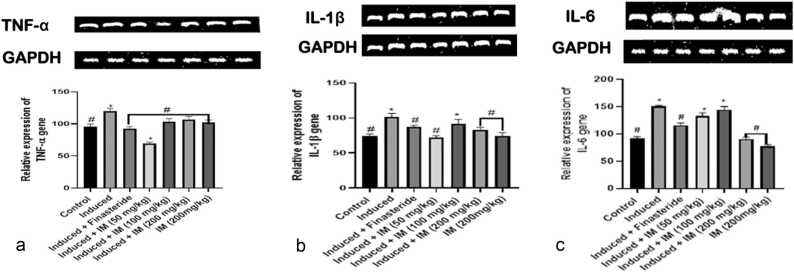

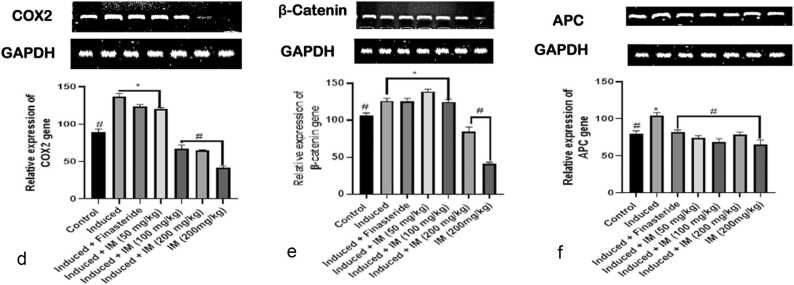
a.: An illustration of the densitometric assessment of the reverse transcriptase polymerase chain reaction (RT-PCR) investigation of TNF-α expression using agarose gel electrophoresis. * and # denote statistical differences to the control group (p < 0.05) and the prostate cancer control group (p < 0.05), respectively. Fig. 4b. The densitometric evaluation of the reverse transcriptase polymerase chain reaction (RT-PCR) study of IL-1β expression on agarose gel electrophoresis is represented in a snapshot. *Denote statistically significant difference from control (p < 0.05), whereas #Denote statistically significant difference from prostate control (p < 0.05). Fig. 4c: A snapshot of the reverse transcriptase polymerase chain reaction (RT-PCR) investigation of IL-6 expression using agarose gel electrophoresis and densitometric assessment. *Denote statistical variation from control (p < 0.05), and #Denote statistical variation from prostate control (p < 0.05). Fig. 4d. An illustration of the densitometric assessment of the reverse transcriptase polymerase chain reaction (RT-PCR) measurement of COX2 expression using agarose gel electrophoresis. *Show significant differences from the control (p < 0.05), whereas #Show statistical differences from the control for prostate cancer (p < 0.05). Fig. 4e A snapshot of the reverse transcriptase polymerase chain reaction (RT-PCR) investigation of β catenin expression using agarose gel electrophoresis and densitometric assessment. *Show significant differences from the control (p < 0.05), whereas #Show statistical differences. Fig. 4f An illustration of the densitometric assessment of the reverse transcriptase polymerase chain reaction (RT-PCR) study of APC expression using agarose gel electrophoresis. *Show the statistical difference from the control group (p < 0.05), and #Show the statistical difference from the prostate group (p < 0.05).

When *I. macrophylla* is administered orally, the results demonstrate a significantly increased expression (p < 0.05) of TNF-α in the induced groups, but not relatively expressed in the control group (Fig. 4a). Compared to the control group, groups treated with *I. macrophylla* at 100 and 200 mg/kg showed an upregulation of TNF-α (p < 0.05). The effects of given *I. macrophylla* extract on pro-inflammatory cytokines such as IL-1β in the pancreas of rats with PCa caused by cacodylic acid are shown in Fig. 4b. Notably, compared to the control and treatment groups, the induced group showed over-expression of IL-1β. However, a significant down-regulation of IL-1β was observed in treatment groups compared to the prostate-induced group. Our study indicates that *I. macrophylla* at higher doses significantly reduced IL-6 expression (Fig. 4c). The activity of *I. macrophylla* against COX2 gene expression was also investigated in this study. We found that at moderate and higher dosages of 100 mg/kg and 200 mg/kg administration, the expression of COX2 gene in the experimental animals was significantly ameliorated (Fig. 4d). Interestingly, our result shows that *I. macrophylla* appears to antagonise COX2 expression unlike the standard drug, indicating that *I. macrophylla* is likely more efficient in its anti-PCa activity, even though at moderate and higher dosages. Oral administration of *I. macrophylla* effected a significant over-expression (*p* < 0.05) of β-Catenin in rats induced prostate cancer but not relatively expressed in the control group. However, the outcome is different for β-Catenin, where only the highest dosage (200 mg/kg) of administration effected a significant reduction in the expression level of the gene in PCa-induced animals. The expression of the β-Catenin gene was significantly upregulated (*p* < 0.05) in groups treated with *I. macrophylla* (100 mg/kg) and (200 mg/kg) relative to the control group. Moreover, our study shows that at all dosages of administration of *I. macrophylla* in PCa-induced rats, a significant reduction in the expression level of APC was manifested compared to the control group (Fig. 4f).

### 3.6 Effect of oral administration of *I. macrophylla* on apoptotic genes

**Fig. 5:**
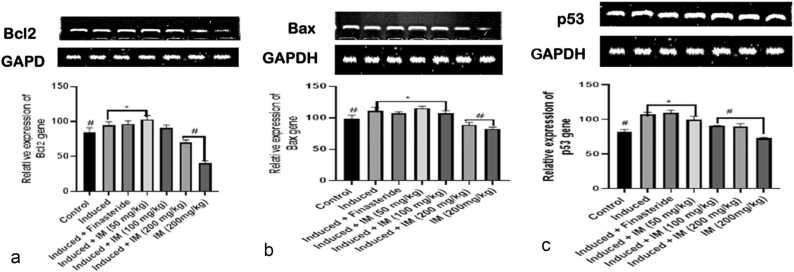

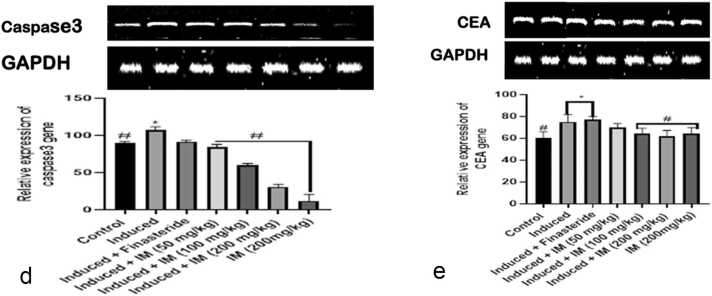
a. A snapshot of the densitometric assessment of the agarose gel electrophoresis for the Bcl2 expression analysis using reverse transcriptase polymerase chain reaction (RT-PCR) study. *Show significant differences from the control (p < 0.05), whereas #Show statistical differences from the control for prostate cancer (p < 0.05). Fig. 5b.RT-PCR (reverse transcriptase polymerase chain reaction) study of Bax expression using agarose gel electrophoresis and densitometric assessment in a snapshot format. *Show the statistical difference from the control group (p < 0.05), and #Show the statistical difference from the prostate group (p < 0.05). Fig. 5c: A snapshot of the densitometric assessment of the agarose gel electrophoresis for the P53 expression study using the reverse transcriptase polymerase chain reaction (RT-PCR). *Show the statistical difference from the control group (p < 0.05), and #Show the statistical difference from the prostate group (p < 0.05). Fig. 5d. A snapshot of the reverse transcriptase polymerase chain reaction (RT-PCR) study of caspase3 expression using agarose gel electrophoresis and densitometric assessment. *Show the statistical difference from the control group (p < 0.05), and #Show the statistical difference from the prostate group (p < 0.05). Fig. 5e: An illustration of the densitometric assessment of the reverse transcriptase polymerase chain reaction (RT-PCR) analysis of CEA expression using agarose gel electrophoresis. *Show significant differences from the control (p < 0.05), whereas #Show statistical differences from the control for prostate cancer (p < 0.05).

The effects of *I. macrophylla* administration on the Bcl2 and BAX genes of rats given cacodylic acid-induced PCa are depicted in Fig. 5a and b. Notably, the induced group showed higher levels of BAX expression than the control and treated groups. However, in contrast to the prostate-induced group, a notable downregulation of BAX was noted in the therapy groups. Our result shows that Bax is upregulated at the administration of 200 mg/kg of *I. macrophylla* extract, while Bcl2 was upregulated at the highest dosage (200 mg/kg) investigated, thus significantly reducing PCa proliferation at this dosage. The results also show that the p53 gene was expressed more at the 100 mg/kg and 200 mg/kg administration of *I. macrophylla* extract (Fig. 5c), thus serving as an anti-cancer proliferative agent at these concentrations, respectively. In addition, there was an indication that at the effective dosages of administration (100 mg/kg and 200 mg/kg), this extract is more effective against cancer cells, unlike the standard drug, depicting the possibilities of its potent specificity beyond the standard treatment. Moreover, we observed that at all the dosages (50 mg/kg, 100 mg/kg and 200 mg/kg) of *I. macrophylla* extract investigated against PCa in rats, there was a significant reduction (P<0.05) in the spread of cancerous cells in the experimental animals, suggesting that the extract upregulated caspase 3 gene at all levels of dosage administration (Fig. 5d). Our study shows that *I. macrophylla* administration at 100 mg/kg and 200 mg/kg concentrations significantly (p<0.05) reduced CEA levels in PCa induced rats (Fig. 5e). This significant reduction was not seen with the 50 mg/kg extract administration.

## 4 Discussion

The main objective of this study is to investigate the anticancer property of *I. macrophylla*, which has not been studied previously. According to Table 2, our work confirms earlier research showing *I. macrophylla* to be a rich source of bioactive components such as phenols, flavonoids, isoflavonoids, terpenes, phytosterols, quinones, and glycosides. These substances are part of the plant's therapeutic qualities and may be used to treat a number of illnesses [27], [28], [29], [30]. These bioactive components may have been responsible for the polygenic anticancer activity observed in this study. However, further investigations are needed to fully explore the therapeutic potential of this diverse genus.

As compared to the control group, the prostate weight of the PCa-induced group was considerably higher, according to our results (Table 3). In contrast to the untreated control, the high dose of *I. macrophylla* given to the treated group considerably reduced prostate weight. Rats receiving conventional treatment (finasteride) had a 59.43 % reduction in prostate weight compared to the without-treatment control group. The groups that received *I. macrophylla* had dose-dependent weight decreases (50, 100, and 200 mg/kg) and showed reductions of 48.85 %,.60 %, and 70.29 %, respectively. Research has revealed conflicting theories on the relationship between prostate weight and the onset of PCa. The idea that prostate weight raises the risk of prostate cancer has been refuted by recent research. Strong evidence of a negative correlation that exists between prostate weight and PCa incidence was discovered in a recent thorough review [31]. It is thought that the mechanical stress brought on by a growing transition zone, which causes fibrosis and atrophy in the outermost area where the majority of PCa originates, accounts for the protective role of larger prostates against PCa [32]. These results are contrary to past research, especially in animals, including this present study, where a positive correlation between prostate weight and cancer risk was demonstrated. However, it's crucial to remember that using transgenic mice and other animal models has helped researchers understand how PCa progresses and create viable treatment plans [33]. Studies on the correlation between prostate weight and PCa development in animal models vis-à-vis human subjects deserve more research.

**Table 3:** Effect of *I. macrophylla* leaf extract on body weight, prostate wet weight and prostate index of rats.

Groups	Initial wt. (g)	Final wt.(g)	Diff in wt.(g)	Prostate wt. (g)	Prostate Index (PI)
Control	241.0	252.5	11.5	0.88±0.16	0.35±0.04
Control + IM	218.0	226.6	8.67	0.67±0.19	0.30±0.05
Induced	171.6	244.2	72.6	1.75±0.23	0.72±0.04
Finasteride	158.5	169.3	10.7	0.71±0.21	0.42±0.05
Induced + 50 mg/kg b.wt	179.8	192.0	12.2	1.00±0.17	0.52±0.04
Induced + 100 mg/kg b.wt	177.5	189.8	12.3	0.70±0.12	0.37±0.05
Induced + 200 mg/kg b.wt	155.8	167.5	11.7	0.52±0.18	0.31±0.04

According to recent investigations, the TNF-α gene is overexpressed in PCa tissues and cell lines. A protein involved in inflammation and immunological response is encoded by the TNF-α gene [34], [35]. According to our research, *I. macrophylla* extract administered at any dosage effectively activates TNF-α, just like the conventional medication. The involvement of tumor necrosis factor-alpha (TNF-α) in PCa development is multifaceted. PCa risk has been linked to genetic variations in the TNF-α gene [36]. A different study found that TNF-A -1031C-863C-857T-308G and TNF-α-1031 CC genotype and the haplotype expressions were associated with a higher risk of prostate cancer [37]. The TNF-α-238, an allele, indicated a substantial risk for prostate cancer. However, an investigation found no statistically significant connections [38]. In the biology of prostate cancer, TNF-α has contradictory roles. It can promote castrate resistance and stimulate tumor angiogenesis but can also prevent neovascularization and cause cancer cells to die [39]. These contradictory outcomes underscore the intricacy of TNF-α's role in the initiation and advancement of prostate cancer.

According to our research, *I. macrophylla* considerably decreased IL-6 expression at higher doses. The IL-6 gene is overexpressed in PCa tissues and cell lines [40], which increases the production of IL-6 protein and promotes tumour growth and progression [41]. Elevated IL-6 levels are associated with hormone-refractory and metastatic prostate cancer [42]. Indeed, MAPK, JAK/STAT3, and PI3K are only a few of the signalling pathways that are activated by IL-6, and these pathways support the formation of tumours, have anti-apoptotic properties, and preserve phenotypic tumour progenitor cells [43]. Similar to our study, IL-6 expression was linked to tumour stages and levels of prostate-specific antigen (PSA) [44]. Comparing the administration of *I. macrophylla* to the standard medication revealed that an increased dose of *I. macrophylla* extract (200 mg/kg) is required to reverse the expression and activity of IL-6 in the animals under investigation. Another study [45] reported that small-molecule inhibitors of IL-6 signalling decreased the development and proliferation of prostate cancer cells, which is consistent with our findings. Therefore, targeting *I. macrophylla* against the IL-6 beta gene might constitute a potential therapeutic approach for the treatment of prostate cancer.

Administering *I. macrophylla* extract at medium and higher dosages of 100 mg/kg and 200 mg/kg considerably reduces the expression level of COX-2 in experimental animals. According to recent research, COX-2 expression is elevated in PCa patients compared to healthy controls, contributing to resistance to radiation and chemotherapy in PCa cells [46]. Cox-2 has also been linked to an unfavourable prognosis [47] COX-2 enzymes are partly responsible for producing prostaglandins, which are pro-inflammatory chemicals that stimulate tumour growth and angiogenesis [48]. It's interesting to note that, in contrast to the usual medication, our result for *I. macrophylla* appears to inhibit COX-2 expression, suggesting that the extract has a stronger potential for anti-PCa activity, especially at medium and higher dosages. In agreement with our research, it has been demonstrated that inhibiting COX-2 slows the development of prostate cancer cells and triggers apoptosis [49]. As a result, using organic inhibitors like *I. macrophylla* to target COX-2 may be a useful tactic for treating and preventing PCa.

In contrast, *I. macrophylla* exhibited a characteristic sigmoidal effect on the expression of IL-1β gene in this study. The findings indicate that administering the plant extract at two different dosages—50 mg/kg and 200 mg/kg—significantly reduces the expression of IL-1β, while the medium dosage has no such effect in vivo. IL-1β has been reported in previous studies to exhibit pleiotropic actions on immune cells, angiogenesis, cancer cell proliferation, migration, and metastasis. As such, it is an important androgen-responsive immunotherapeutic target for advanced prostate cancer [50]. Debates to use or not to utilize IL-1β inhibitors in cancer treatment have risen because anti-cancer medicines can also stimulate the synthesis of IL-1β by immune or cancer cells, with differing consequences on the progression of cancer [51]. Our findings are supported by the possibility that genetic polymorphisms in the IL-1β gene, which are linked to a higher risk of aggressive prostate cancer, are the causes of the different expression patterns of IL-1β and its receptors in PCa compared to normal prostate tissue [52]. Certain IL-1β polymorphisms, however, might be protective against the development of PCa. Particularly, a lower incidence of PCa has been associated with IL1β-511(rs16944) AG and IL1β-31(rs1143627) genotypes [53]. These results emphasize the intricate connection between IL-1β and PCa, indicating possible functions in the development and avoidance of cancer. These findings imply that IL-1β can stimulate and repress PCa growth in an inflammatory milieu, hence aiding in its development and survival [54]. More research is required to determine the genetic polymorphism of IL-1β in the presence of extracts from *I. macrophylla*.

Furthermore, our research demonstrates that the administration of *I. macrophylla* in rats with PCa-induced expression of β-Catenin and APC is significantly affected. Only the highest dosage (200 mg/kg) of *I. macrophylla* administration significantly reduced the expression level of β-Catenin in PCa-induced animals. However, a significant reduction in the expression level of APC was observed at all dosages compared to the control group. Research indicates that the APC gene, which is found on chromosome 5q, forms a destruction complex involving GSK-3b, Axin/Axin2, and CK1 to negatively regulate the β-Catenin/Wnt pathway [55]. When there is an APC mutation, β-Catenin builds up, moves to the nucleus, and promotes the transcription of Wnt target genes in gastrointestinal epithelial stem cells, which can lead to cancer [56]. Although there are distinct distinctions in the pathways activated and the tumour morphologies, loss of TGFβ signals in mouse prostate epithelium collaborates with the deletion of either the Apc or Pten cancer suppressor genes to cause invasive PCa [57]. Overexpression of β-catenin in tumor tissues then activates downstream target genes such as c-Myc and cyclin D1, which encourage the proliferation and survival of cancer cells [58], [59]. Furthermore, it has been demonstrated that β-catenin interacts with other signalling pathways, such as the PI3K/Akt pathway, to promote the proliferation, invasion, and survival of PCa cells [60], [61]. Zhang *et al*. [62] reported that prostate cancer cell migration and invasion were decreased by a siRNA targeted at β-catenin, which is consistent with our observations. Thus, overexpression of β-catenin and APC is essential for the formation and advancement of prostate cancer since it promotes cell migration, survival, and proliferation, which in turn aids in tumour growth and metastasis. Therefore, using substances like *I. macrophylla* extract to target β-catenin and APC may be a viable therapy for the treatment of PCa.

Additionally, administering *I. macrophylla* to animals induced with PCa generated statistically significant CEA levels from the control (Fig. 5e). Previous reports show that prostate-specific antigen (PSA) levels and tumor stage were connected with CEA levels, which were found to be considerably higher in PCa patients compared to healthy subjects [63], [64]. Similar to our investigation, CEA knockdown impeded the migration and multiplication of prostate cancer cells [65]. It was also discovered that in mice models, CEA-targeted treatment prevented PCa from growing and spreading [66]. Our findings may thus be a useful tactic for the improvement of prostate cancer (PCa) since CEA overexpression stimulates prostate cancer cell invasion and metastasis [67] and functions as a possible therapeutic target for prostate cancer [60]. Furthermore, we discovered that the p53 gene was overexpressed following the administration of plant extract at doses of 100 mg/kg and 200 mg/kg, respectively, acting as an anti-tumor proliferating agent. According to a study, tumor stage and prostate-specific antigen (PSA) levels were connected with p53 expression [68]. Therefore, one possible treatment approach for prostate cancer is to restore p53 function. Reintroducing functioning p53 into prostate carcinoma cancer cells prevented tumor development and metastasis, which is consistent with our findings. Our result also shows that, at the effective dosages of administration, this extract is more effective against cancer cells, unlike the standard drug, depicting the possibilities of its specificity beyond the standard treatment. This may also be an indication of potent phytochemicals in the plant extract against PCa development and progression. Therefore, utilizing plant-based extracts like *I. macrophylla* to restore p53 function could be a promising therapeutic approach for the treatment of prostate cancer.

Our findings demonstrate that when 200 mg/kg of *I. macrophylla* extract was administered, Bax and Bcl2 expression were increased, which at this dosage considerably reduces PCa proliferation. These could also mean that *I. macrophylla* extract's anticancer properties only work at larger concentrations when it comes to the Bax and Bcl2 pathways. The function of the Bcl2 and Bax family proteins in PCa, as well as their potential for gene therapy, have been investigated in recent research. Although the association is not always significant, Bax expression has been reported to be associated with Gleason score in prostate cancer [69]. Li *et al*. [70], reported that the overexpression of Bax in PCa cells may result in reduced activity of store-operated calcium entry, hence increasing susceptibility to drugs that induce apoptosis. Bax-based gene therapy techniques have demonstrated potential in eliciting bystander death effects in prostate cancer cells in conjunction with other apoptotic molecules such as Fas Ligand and TRAIL [71]. Crucially, regardless of the Bcl-2 or androgen sensitivity of different prostate cancer cell lines, adenovirus-mediated overexpression of Bax has been shown to trigger apoptosis [72]. Furthermore, compared to neighbouring non-cancerous tissues and benign prostatic hyperplasia, Bcl2 gene expression is elevated in PCa tissues [73]. Dysregulation of Bcl2 family members, which control the shift from androgen-dependent to androgen-independent growth, is linked to the emergence of castration-resistant prostate cancer (CRPC) [74]. Inhibition of cell death, particularly through the intrinsic apoptotic pathway governed by Bcl2 proteins, is a key factor in prostate cancer development and therapy resistance [75]. Understanding the intricate interactions between androgen receptor signalling, paracrine factors, and apoptosis regulation is essential for developing effective treatments for advanced prostate cancer [76]. Emerging therapies targeting Bcl2 family proteins, such as selective and pan-Bcl2 inhibitors, can potentially restore cellular death in cancer cells [74]. These findings suggest that Bax- and Bcl2-based therapies could be effective against various stages of prostate cancer, including advanced androgen-independent forms. Restoring the functions of these genes may be a promising therapeutic strategy for PCa treatment.

In rats induced with PCa, the expression of caspase-3 was markedly elevated across all tested dosages of *I. macrophylla* extract. Caspases, essential executioner proteases in apoptosis, have a major influence on the course and outcome of prostate cancer treatment. On the other hand, procaspase-3 is overexpressed in many cancers, which could promote cancer growth [77]. Deregulation of apoptosis contributes to metastasis, androgen insensitivity, and tumor induction in prostate cancer [78]. Effective cancer therapy requires caspase activation, and numerous treatments focus on endoplasmic reticulum stress, extrinsic stress, or intrinsic stress pathways [79]. Normal prostatic secretory epithelial cells have been shown to contain caspase-3, and a changed expression of this protein may be a factor in resistance to medication [80]. Understanding caspase regulation has enabled the development of novel therapeutic approaches for prostate cancer and using procaspase-3 enzymes as targeted anticancer treatments is one such tactic [77], [78].

The photomicrograph of the prostate tissue of the control rat revealed typical convolutions, which are surrounded by two layers of epithelium and clearly demonstrate the basal polarity of the nuclei (group 1). A slightly compromised histoarchitecture coupled with inflammation and necrosis of the prostate tissues was observed in albino rats in the group administered the plant extract without PCa induction. However, group 3 displayed severe blood congestion in their prostate tissue (group 3) as opposed to group 1. In this untreated group, further degeneration was noted as larger acini primarily swollen with secretory contents. Additionally, the prostate tissue in the therapy groups showed enhanced intra-acini papillary convolutions along with back-to-back microacini (groups 5–7). The prostatic dilatation seen in test groups (Fig. 5–7) and standard control (group 4) in comparison to the control (group 1) is suggestive of a general improvement in histopathology. The histopathological alteration seen in groups 1 and 3 (the PCa untreated group) validates the features that [81] and [82] previously reported. The mechanism of action may be the binding of DHT, or additional phytochemicals present in our extract to androgen receptors, which stimulate protein synthesis and prostatic cell proliferation.

## 5 Conclusion

This study demonstrated the modulation of anti-inflammatory and apoptotic genes by the activities of extracts from *I. macrophylla* in the prostate gland of PCa-induced rats. Evidence from our study suggests this plant extract has a vast and enormous potential in ameliorating the activities of multiple genes linked to inflammation and apoptotic activities leading to PCa, as well as selected genes indicated in PCa activation, development, growth, and invasion pathways in experimental animals. Further works will focus on identifying the most active phytochemical and bio-component in the plant extract and specific gene-focused investigation using transgenic experimental animal models. This will, in addition to the present study, provide embellished and more evidence-based facts for the anticancer potential of this plant.

## Ethical approval

Not applicable

## Funding statement

This research received no specific grant from funding agencies in the public, commercial, or not-for-profit sectors.

## Author statement

We, the undersigned, declare that this manuscript is original, has not been published before and is not currently being considered for publication elsewhere. We confirm that the manuscript has been read, reviewed, and approved by all named authors and that no other persons have satisfied the criteria for authorship but are not listed. We further confirm that the order of authors listed in the manuscript has been approved by all of us. We understand that the Corresponding Author is the sole contact for the Editorial process. He is responsible for communicating with the other authors about progress, submissions of revisions and final approval of proofs.

## CRediT authorship contribution statement

**Adedotun Olayemi Oluwatuyi:** Visualization, Formal analysis, Data curation. **Joseph Ashaolu:** Writing – review & editing, Visualization, Software, Formal analysis, Data curation. **David Morakinyo Sanni:** Project administration. **Olusola Olalekan Elekofehinti:** Supervision, Conceptualization. **Gbenga Oluwaseyi Alabi:** Writing – original draft, Methodology, Conceptualization.

## Declaration of Competing Interest

The authors declare that they have no known competing financial interests or personal relationships that could have appeared to influence the work reported in this paper.

## Data Availability

Data will be made available on request.
